# A multi-component, community-engaged intervention to reduce cardiovascular disease risk in perimenopausal Latinas: pilot study protocol

**DOI:** 10.1186/s40814-020-00756-1

**Published:** 2021-01-06

**Authors:** Yamnia I. Cortés, Diane C. Berry, Krista M. Perreira, Alison Stuebe, Lee Stoner, Cheryl Woods Giscombé, Jamie Crandell, Lymarí Santíago, Latesha K. Harris, Mayra Duran

**Affiliations:** 1 School of Nursing, The University of North Carolina at Chapel Hill, Campus Box 7460, Chapel Hill, NC 27599-7460 USA; 2 Department of Social Medicine, The University of North Carolina at Chapel Hill, 333 South Columbia Street, MacNider Hall, Campus Box 7240, Chapel Hill, NC 27599-7240 USA; 3 Department of Obstetrics and Gynecology, Division of Maternal-Fetal Medicine, The University of North Carolina at Chapel Hill, 3010 Old Clinic Building, Campus Box 7516, Chapel Hill, NC 27599-7516 USA; 4 Department of Exercise and Sport Science, The University of North Carolina at Chapel Hill, 306 Woollen Gym, Campus Box 8605, Chapel Hill, NC 27599-8605 USA; 5 Department of Biostatistics, Gillings School of Global Public Health, The University of North Carolina at Chapel Hill, Campus Box 7460, Chapel Hill, NC 27599-7460 USA

**Keywords:** Cardiovascular disease, Hispanic/Latino, Menopause, Health behavior, Self-efficacy, Stress

## Abstract

**Background:**

Cardiovascular disease (CVD) risk increases substantially during perimenopause. Latinas have a significantly worse CVD risk factor profile than non-Hispanic White women, potentially due to multiple sociocultural and environmental factors. To date, interdisciplinary interventions have not focused on improving nutrition, physical activity, stress management, and biologic CVD risk in perimenopausal Latinas. The purpose of this study is to examine the feasibility and initial efficacy of a multi-component intervention to reduce CVD risk in perimenopausal Latinas.

**Methods:**

This is a two-group, repeated measures experimental study. Eighty perimenopausal Latinas (age 40–55 years) from two community groups will be randomized: one group will complete the intervention; the other will be a wait-list control. The intervention consists of 12-weekly sessions (education, physical activity, stress management, coping skills training), followed by 3 months of continued support, and 6 months of skill maintenance on their own. The primary outcomes include arterial stiffness, blood pressure, lipids, and blood glucose. Secondary outcomes are health behaviors (nutrition, physical activity, sleep, coping strategies), self-efficacy, and other biological factors related to CVD risk (adiposity, C-reactive protein, hair cortisol, vasomotor symptoms). We will assess changes in outcomes from Time 1 (baseline) to Time 2 (6 months) and Time 3 (12 months) using general linear mixed models to test the hypotheses. We will also evaluate the feasibility of the intervention by assessing enrollment and retention rates, barriers, and facilitators to enrollment, intervention fidelity, the suitability of study procedures, and participant satisfaction with the intervention and study protocol. We hypothesize the intervention group will decrease biologic CVD risk and improve health behaviors and self-efficacy significantly more than the wait-list control.

**Discussion:**

Results from this study will contribute to knowledge on the feasibility of behavioral interventions, including stress management and coping skills training, which could reduce CVD burden among perimenopausal Latinas. Because Hispanic/Latinos are the largest ethnic minority in the United States (US), progress regarding CVD risk among perimenopausal Latinas may lead to significant improvement in the overall CVD burden in the US.

**Trial registration:**

Prospectively registered, NCT04313751 (03/19/2020), Protocol version 1.0

## Background

Cardiovascular disease (CVD) remains the leading cause of death for women globally [1]. Hispanic women (Latinas) in the United States (US) have a significantly worse CVD risk factor profile than non-Hispanic White women, including higher rates of diabetes, uncontrolled hypertension, and metabolic syndrome [2, 3]. Also, the prevalence of coronary heart disease is higher among Latinas (6.1%) than non-Hispanic Black (5.7%), and non-Hispanic White (5.3%) women [4]. Despite being at greater risk for CVD, only 34% of Latinas are aware that CVD is the leading cause of death in women [5]. While evidence-based guidelines promote healthy lifestyle behaviors to reduce CVD risk [6], Latinas remain considerably less likely to meet physical activity and dietary guidelines than non-Hispanic White women, particularly during midlife [7].

The risk of CVD increases substantially during perimenopause [8–11], a critical period of physiologic changes, which ranges from 6–8 years before the final menstrual period [12]. Perimenopause has been associated with an increase in body mass index, blood pressure, and adverse lipid profiles [9, 13]. Among Latinas, the Hispanic Community Health Study/Study of Latinos found that the age-adjusted prevalence of metabolic syndrome, a risk factor for CVD [14], ranged from 35 to 55% in women age 40–59 years, compared to 10–26% in women less than 40 years [15]. Arterial stiffness, a measure of “vascular aging” [16] often predictive of CVD [17], may provide information beyond standard CVD risk factors alone [18]. Importantly, arterial stiffness measured by carotid-femoral pulse wave velocity (cfPWV) has been shown to increase during perimenopause [11]. Similarly, menopause is associated with elevated levels of proinflammatory cytokines [19, 20] and C-reactive protein [21], thought to play a role in the development of arterial stiffness [22, 23].

Multiple sociocultural and environmental factors such as lower socioeconomic position, discrimination, and psychological stress are associated with elevated CVD risk [24]. These factors are related to CVD through their influence on health behaviors [25, 26] as well as potential mechanisms involving inflammatory and neuroendocrine pathways [27–30]. Latinas in the Study of Women’s Health Across the Nation, a multi-site epidemiologic study of women’s midlife health, reported higher levels of perceived stress than perimenopausal women of any other racial/ethnic group [31]. In 2009, 36% of Hispanics/Latinos in the US lived in high-poverty environments [32]. Living in disadvantaged and unsafe neighborhoods is associated with adverse cardiovascular risk factors, including increased stress [33] and reduced access to healthy foods [34]. Additionally, Latinas face many environmental barriers that limit their physical activity, including crime, traffic, lack of recreational facilities, and concern about deportation [35, 36]. A more favorable neighborhood social environment (i.e., greater social cohesion and safety) has been associated with longer sleep duration [37]. Over half of perimenopausal Latinas report difficulty sleeping [38], which has been associated with arterial stiffness [39].

Prior lifestyle interventions among Hispanics/Latinos have been successful in increasing knowledge of CVD risk, physical activity, and a heart-healthy diet [40–43]. Many of these interventions have incorporated an evidence-based community health worker (CHW)-led curriculum—*Su Corazón, Su Vida* (SCSV) [41, 44]—a 12-lesson curriculum consisting of information on CVD risk awareness, healthy food choices, and physical activity [45]. Cultural adaptation of interventions can range from adaptation of language and setting to adaptations that use a within culture perspective to include culturally congruent concepts and goals [46, 47]. Importantly, *SCSV* is a bilingual (English, Spanish) curriculum that integrates culturally tailored activities and handouts with heart-healthy messages to engage Hispanics/Latinos in cardiovascular health promotion. The curriculum has been used among Latinos age ≥ 18 in the US and Mexico, successfully improving health behaviors [41, 48, 49]. Earlier studies incorporating *SCSV* have largely focused on group and individual education sessions [49, 50].

Clinical trials among younger Latinas have shown that effective coping strategies can improve self-efficacy and health behaviors [51]. Coping skills training is a cognitive-behavioral intervention that focuses on social problem-solving, cognitive restructuring, assertiveness training, and conflict resolution to improve self-efficacy [52, 53]. In addition to coping skills training, the American Psychological Association suggests implementing stress reduction interventions for Hispanics/Latinos that focus on recognizing and managing stressors and accessing resources to minimize stress exposure [54]. To date, we have found no interventions that have been developed to help perimenopausal Latinas reduce their risk of CVD using a culturally and language-tailored intervention. We propose to assess the feasibility and initial efficacy of a multi-component behavioral intervention integrating the *SCSV* curriculum and coping skills training [53] with physical activity and stress management. We will continue to provide support as women increase their self-efficacy and practice new health behaviors. The aims of this study are as follows:

1. To examine the feasibility of the multi-component behavioral intervention. We will assess enrollment and retention rates; barriers and facilitators to enrollment; intervention fidelity; suitability of study procedures and outcome measures; and participant satisfaction with the intervention and study protocol.
2. To evaluate the initial efficacy of the intervention by comparing an intervention group to a wait-list control group in a pilot study from Time 1 (baseline) to Time 2 (6 months) and from Time 1 (baseline) to Time 3 (12 months). We will assess whether the intervention (a) decreases biological CVD risk factors (arterial stiffness, blood pressure, lipids, blood glucose), (b) improves health behaviors and self-efficacy, and (c) improves adiposity, inflammatory and stress biomarkers, and vasomotor symptoms.

### Theoretical framework

**Fig. 1:**
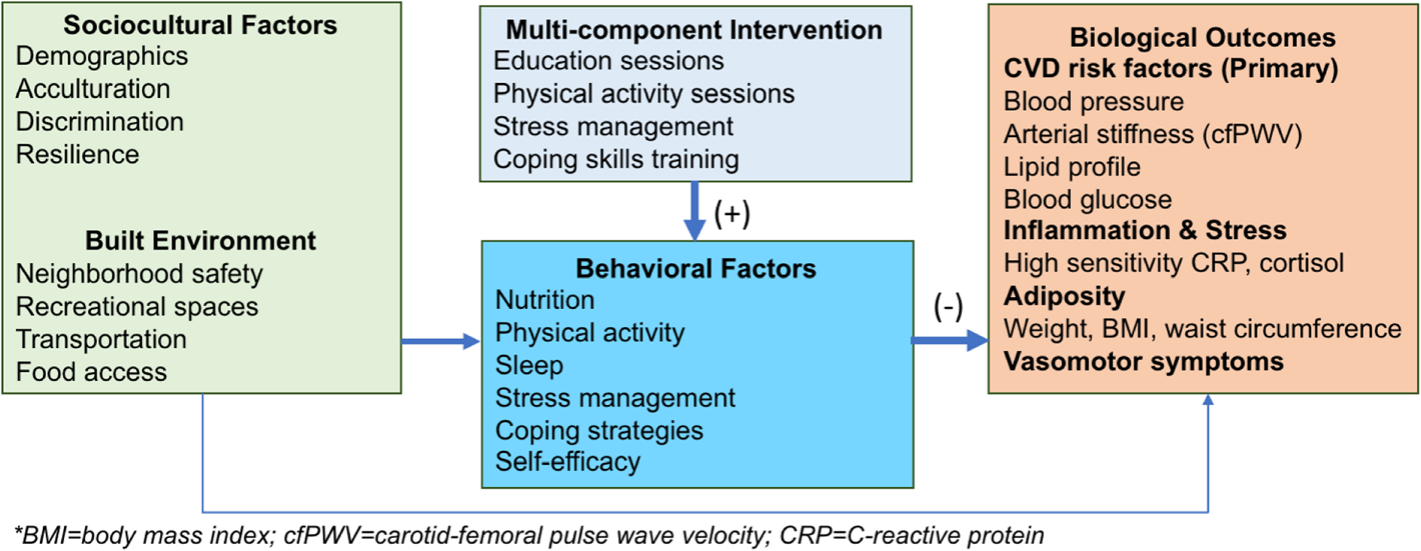
Conceptual framework

This study is guided by social cognitive theory [55–57] and the socioecological model [58]. Figure 1 depicts our conceptual framework.

Social Cognitive Theory posits that enhancing an individual’s knowledge and skills to perform a new behavior improves self-efficacy, which in turn increases the likelihood that the new behavior will be maintained. This study will measure the self-efficacy of several behaviors, including eating, physical activity, and coping. Studies have suggested that an individual’s perceived ability to cope effectively with a problem can enhance the individual’s ability to address the next problem and enact healthy behaviors [53, 59].

The socioecological model considers the complex interplay between individual-level factors, interpersonal relationships, the built environment, and sociocultural environment [58]. Using this model, we posit that an individual’s behavior, such as physical activity, is influenced by a lack of safe recreational spaces in the built environment or a sociocultural environment of poverty that limits resources to seek opportunities for physical activity. An obvious implication of this interaction is that improving the sociocultural and built environment will benefit communities and individuals. While the current intervention does not directly address this larger context, it will provide women peer support and information on how to improve their health within the context of their everyday lives.

## Methods/design

### Study design

**Fig. 2:**
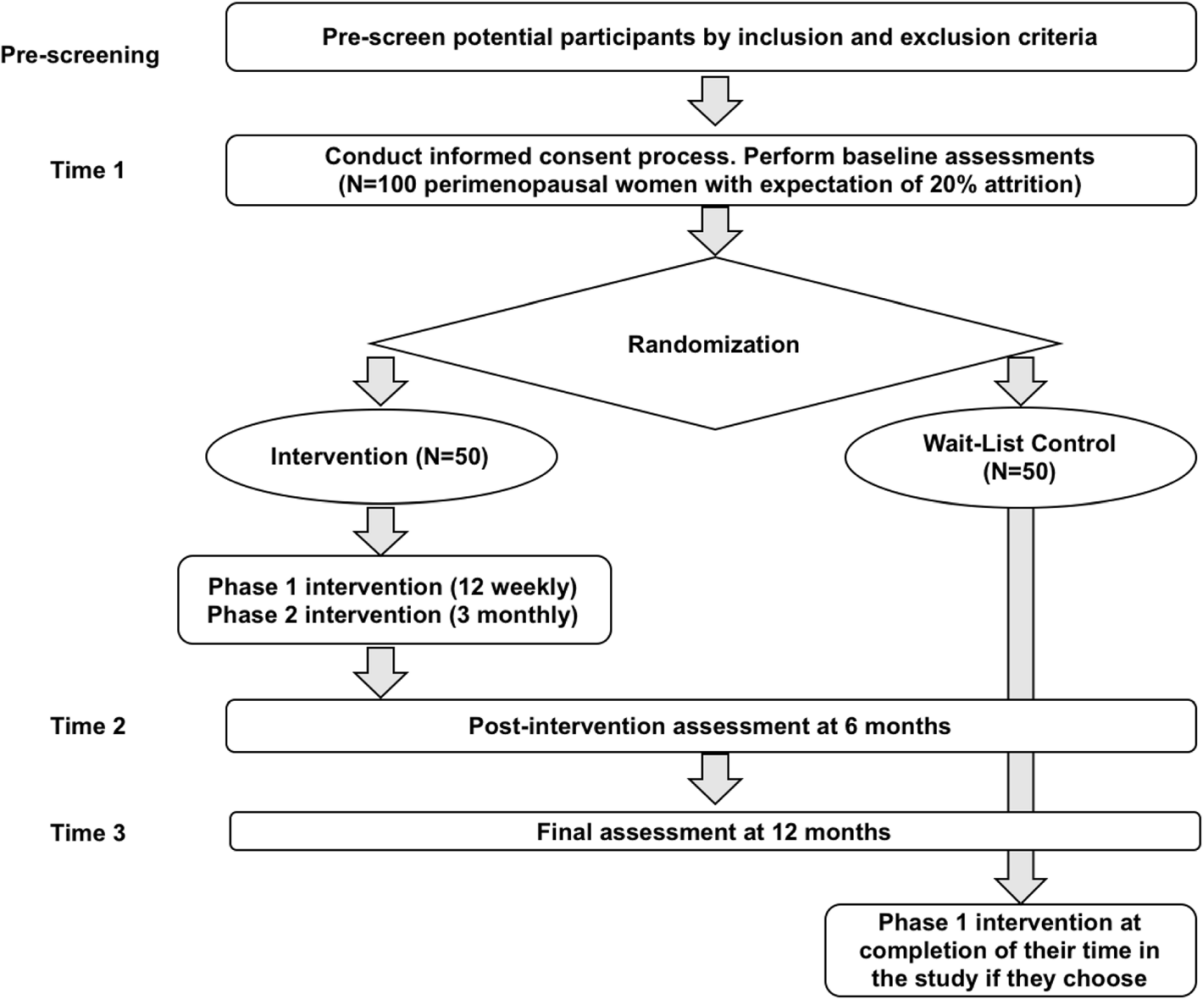
Study flow diagram

This pilot study will use a two-group, randomized repeated measures study design to evaluate the intervention’s initial efficacy with 80 perimenopausal Latinas (Fig. 2). Participants will be recruited from two local churches and community centers providing programs and services to Latinas. After baseline assessment (Time 1), we will randomly select one of the two churches to receive the intervention; the other will be assigned to a wait-list control. The intervention group (*n* = 40) will receive a 3-phased intervention. In Phase I (Intensive Intervention), CHWs will meet with women in small groups (8–10 women) to deliver a 12-week intensive intervention including 45 min of cardiovascular health education using the *SCSV* curriculum and coping skills training, a 60-min physical activity session, and a 15-min stress management activity (e.g., breathing techniques, mindfulness exercises). Participants will receive handouts to reinforce their learning, including home-based exercises and recipes. Phase II (Continued Support) will consist of three monthly 60-min sessions led by CHWs to problem-solve issues related to nutrition and exercise and stress management and to provide feedback and support. During Phase III (Follow-up), participants are expected to maintain skills on their own for 6 months.

Data will be collected at Time 1 (0 months [baseline]), Time 2 (6 months [completion of the intervention]), and Time 3 (12 months [after 6 months with no contact from the study staff]). Time 2 data will provide pilot data on the potential effect of the intervention after the intensive intervention and continued support. Time 3 data collection will test the initial efficacy after the participants have had sufficient time to implement their new health behaviors and coping skills on their own. These times were selected because 6 to 12 months after completion of an intervention represents a standard interval for follow-up in interventions during perimenopause [60, 61].

To determine feasibility, this study will include in-depth exit interviews with participants at Time 3, as well as process evaluations and assessment of screening, enrollment, and attendance logs. Data in the wait-list control group (*n* = 40) will be collected at the same time intervals as the intervention group. After Time 3 data collection, they will be offered the Phase I intervention (12 weekly sessions). Both groups will receive a monthly thank you card for their continued participation.

### Study setting and recruitment

The study will build upon established partnerships between The University of North Carolina at Chapel Hill, local churches, and community centers in Orange County and Durham County. Bilingual research assistants (RAs) will be available after church services, at community centers, community health fairs, and at cultural events to distribute brochures, give potential participants information about the study, answer questions, and enroll perimenopausal women. The brochures will describe the purpose of the study, eligibility, length of participation, and the benefits of good nutrition and physical activity. If women are interested, but do not have time to complete screening during a recruitment event, the RAs will privately record their name and telephone number and schedule them for an enrollment screening telephone call. Flyers with information about the study will also be posted at churches and community centers. Flyers will contain a phone number for individuals to call if they are interested in participating. This approach worked well in previous studies [62].

We will rent space for the intervention from two churches, one in Orange County and one in Durham County, with an active Latino ministry, Spanish services, and classroom space. The churches are located in the communities where the participants live and are within walking distance from their homes or on bus lines, thus increasing the chances of successful recruitment and retention. To minimize contamination bias, after enrollment and baseline assessment, we will randomly assign one church to the intervention and the other to the wait-list control group.

### Inclusion criteria, enrollment, and procedure for retention

Inclusion criteria for women in this study are age 40–55 years; self-identification as Hispanic/Latina; ability to understand spoken English or Spanish; perimenopausal (change in length of bleeding or inter-bleeding interval, or no menstrual bleeding in the past 3–11 months) [63]; intact uterus and at least one ovary; not currently pregnant; no hormone therapy or birth control pills in the past three months (as these may alter bleeding patterns). Participants will complete a health history questionnaire to ascertain if they have a heart murmur, congenital heart disease, family history of sudden death, difficulty exercising, or psychological disorder that would prevent their participation. Women will be excluded if they have a history of CVD (heart attack, stroke, coronary heart disease), heart murmur, congenital heart disease, family history of sudden death, or difficulty exercising. Women will not be excluded if they are currently on anti-hypertensive, lipid-lowering, or diabetes medications.

Participants interested in the study will complete a private face-to-face screening with a bilingual RA at the time of recruitment, or a private phone screening. During the screening, RAs will ask women for their birthdate, last menstrual period, the regularity of menstrual periods, history of hysterectomy or oophorectomy, use of hormone therapy or birth control pills, and history of CVD. Women who meet the study criteria will be scheduled for a baseline visit to confirm eligibility, review the study, requirements of participants, the risks and benefits of participating, and random assignment; and all questions will be answered. We plan to enroll 8-10 women per week and can extend the timeline for enrollment if needed. If we have difficulty enrolling participants, we will ask community leaders from the recruitment sites for advice.

To strengthen retention, we will hire bilingual (Spanish/English speaking) staff, and all recruitment and intervention materials and instruments will be available in English and Spanish.

Intervention sessions will be interactive, with culturally tailored content developed to engage and sustain participants’ interest. Women who miss a learning session will be offered an opportunity to review the weekly class content over the phone and will be reminded when the next class will be held. We will provide women with a list of resources and recipes, and handouts in Spanish on how to improve their nutrition, physical activity, and sleep. We will ask participants for phone numbers of family members and permission to call them if we cannot contact them. We will be flexible in scheduling enrollment and data collection appointments and send thank you cards. We will remind participants of their data collection visits one week before and confirm the day before the appointment. These approaches have been successful in prior studies with Latinas, with < 20% attrition [64, 65]. We will make every effort to keep our attrition lower than 20%; if attrition exceeds 20%, we will contact the participants to ask why they stopped coming and develop strategies to assist them in continuing the study if they wish. We will also meet with our Community Advisory Board quarterly to develop and implement strategies to improve retention.

### Ethics and informed consent

All study procedures were approved by The University of North Carolina at Chapel Hill Institutional Review Board. All study materials, including the consent forms will be available in English and Spanish. The RAs (all fluent in Spanish) will complete the informed consent. The Principal Investigator will train research staff about the consenting process. In addition, all research staff will complete Good Clinical Practice and Human Subjects Research training. Women will be assured that their decisions about participation (yes or no) will in no way affect their relationship with community sites, any health facility, or The University of North Carolina at Chapel Hill.

### Sample size and power

Eighty women are expected to complete the study (40 per group). Prior clinical trials among Latinas in North Carolina have found ≤ 20% attrition [62, 65]; therefore, we will enroll 100 women to take a 20% attrition rate into account. After a group of women is enrolled, they will be randomly assigned using a computer to the intervention group or the wait-list control group. This pilot study is effectively a cluster-randomized trial with two groups. Because we have two groups, the pilot is not sufficient to estimate the intraclass correlation of change in outcomes, so we do not use traditional analysis methods for cluster-randomized trials. However, we will be able to use baseline data to estimate potential variability between clusters. We intend to determine the effect size for each outcome addressed in Aim 2 in separate multivariate models. While arterial stiffness is a primary outcome, prior interventions among perimenopausal women have not targeted PWV. Therefore, we calculated power using data from an exercise intervention among perimenopausal women [66]; this study observed a change in systolic BP of 6 mmHg (SD ±12); our sample of 80 participants yields 60% power to detect a change this large and 80% power to detect a change of 5 mmHg (SD ± 8).

### Intervention

#### Intensive intervention (Phase I)

Trained bilingual (English, Spanish) CHWs will deliver learning sessions and 15-min stress management sessions in groups of 20 participants. The intensive intervention consists of 12 weekly sessions that include 60 min of interactive learning, 45 min of physical activity, and a 15-min stress management session. The first 8 sessions will integrate education from *SCSV* related to CVD risk awareness, healthy food choices, physical activity, blood pressure control, and diabetes (Table 1). *SCSV* is a culturally adapted curriculum incorporating culturally specific elements into each education session (e.g., familism) [67]; learning sessions highlight the importance of a heart-healthy lifestyle to enhance the lives of the family and not just an individual family member. *SCSV* uses delivery methods that were modified to account for the learning needs and preferences of Latinos (handouts, recipes, role playing, picture cards, group discussions, novelas), particularly Mexicans [50, 68], the predominant Latino sub-group in North Carolina [69]. All curriculum materials feature Hispanic/Latino women or Latino families engaging in heart-healthy activities. At the end of each session, participants will develop a “weekly goal” to apply what they learned and make a change in their everyday life to improve a health behavior. To enhance the curriculum’s relevance to perimenopause, we will include messages from the American Heart Association Go Red Por Tu Corazón and The Heart Truth for Women [70, 71]. Each week CHWs will give participants a *SCSV* or The Heart Truth handout with information on healthier meals and increasing daily physical activity. In the remaining four learning sessions of Phase I, we will integrate previously tested and acceptable coping skills training sessions (problem-solving, cognitive restructuring, conflict resolution, and assertiveness training) [72, 73] with a focus on barriers for perimenopausal Latinas.

**Table 1:** Overview of interventions sessions

Session	Learning session (60 min), physical activity (45 min), stress management (15 min)	Handouts and activities
**PHASE I: 12 weekly sessions**		
**1**	ILS: Are you at risk for heart disease?	A Day with the Ramirez Family
	Stress management: Awareness mindfulness exercise	
**2**	ILS: Say Yes to Physical Activity	Illustration of stretches and how to be physically active; Sample walking program
	Stress management: Breathing techniques	
**3**	ILS: Help your heart: Control your blood pressure	Places to get a free blood pressure checks; Blood pressure log; Mariana’s food choices
	Stress management: Music/dance	
**4**	ILS: Keep your cholesterol in check	Reading food labels and alternative recipes
	Stress management: Mindful stretches	
**5**	ILS: Aim for a health weight	Tips to help control your weight; Healthy serving sizes; Reading food labels
	Stress management: Mindful eating	
**6**	ILS: Screening and understanding blood glucose; Healthier food choices to prevent diabetes; Stress management topic	Places for glucose checks; “Am I at risk of type 2 diabetes?”
	Stress management: Body scan meditation	
**7**	ILS: Make heart-healthy eating a family affair	Sample menus; Tips for busy families
	Stress management: Positive self-talk	
**8**	ILS: Eating healthy when time and money are tight	Grocery list; Tips to save time and money; Tips for eating out heart-healthy
	Stress management: Adult coloring	
**9**	CST: Increasing physical activity (cognitive restructuring)	Fun physical activities
	Stress management: Awareness mindfulness exercise	
**10**	CST: Understanding barriers to healthy choices, physical activity, and getting enough sleep (problem-solving)	Heart-healthy lottery; Tips for getting enough sleep
	Stress management: Breathing techniques	
**11**	CST: Motivating yourself in a positive manner and getting back on track after a relapse (assertiveness training)	Tips on how to motivate yourself to be physically active daily
	Stress management: Music/dance therapy	
**12**	CST: Working through conflict (conflict resolution)	Lightweights and stretch bands for home use
	Stress management: Mindful walking	
**PHASE II: Three monthly sessions to problem-solve and continued support**		
**PHASE III: Six months of maintenance on own**		

*Note: ILS* Interactive learning session, *CST* Coping skills training

Physical activity classes will be held for 45 min after the interactive learning sessions and will include a warm-up and then activities such as Zumba, Kick Boxing, walking, use of light weights and stretch bands, and a cool-down. These activities were selected due to their acceptability in prior physical activity interventions among Latinas [51, 62, 72]. A bilingual physical activity interventionist certified by the American College of Sports Medicine will teach the physical activity classes and reinforce ways to increase physical activity and decrease sedentary behaviors. At baseline, we will give intervention participants a pedometer, and the RA will train them in its use as part of the intervention. The intervention group will be encouraged to increase their physical activity weekly by small increments until they are averaging 10,000 steps a day or 150 min per week. In addition, the intervention group will be encouraged to develop their own home-based physical activity program using suggestions given in the physical activity session and home-based physical activity handouts. Handouts will also be provided with information on the sociocultural and built environments such as a list of grocers where they can access healthy affordable food and free or low-cost spaces to exercise (e.g., YWCA/YMCA) close to home.

A 15-min stress management activity will be conducted following physical activity sessions. Stress management activities will review various techniques to reduce stress, including deep breathing [74], music therapy [75], mindfulness, and yoga [76, 77]. Missed physical activity sessions will not be made up, but participants can make up learning and stress management sessions.

#### Continued support

During continued support, participants will return to the church for classes once a month for 3 months. As a part of the intervention, participants will engage in a discussion run by the bilingual CHW, who will help women solve problems they have encountered related to nutrition and physical activity for 60 min and then receive a 45-min physical activity class and 15-min stress management session. If a participant misses a class, the bilingual interventionist will call and ask how the woman is doing and give the date of the next class. Continued support classes will not be made up.

### Wait-list control group

A wait-list control group will have data collected at the same time intervals as the intervention group (Time 1, Time 2, Time 3). Participants in both groups will receive up to $135 after the baseline visit, $40 after the 6-month visit, and $45 after the 12-month data collection. Transportation vouchers and childcare will be provided. After participants complete the Time 3 data collection, they will be offered the intensive intervention (Phase I), consisting of interactive learning sessions, physical activity sessions, stress management, and coping skills training, and receive a pedometer. During the course of the study, the wait-list control group will receive monthly cards to thank them for their continued participation in the study and to remind them when they will be eligible to receive the intervention.

### Fidelity of the intervention

We will assess, monitor, and enhance intervention fidelity across five domains: study design, provider training, delivery, receipt, and enactment [78]. For study design, the intervention and control group will have data collected at the same time intervals, and each group will receive the intensive intervention (Phase I). To ensure fidelity, the Principal Investigator and Lead RA will train CHWs to deliver the intervention using a standardized manual. For intervention delivery, the Lead RA will observe two randomly selected sessions per month using a checklist to score delivery based on pre-identified content; we define fidelity as delivering > 80% of the protocol content. If drift occurs, the Principal Investigator will retrain CHWs until the protocol is followed consistently. CHWs will collect data on attendance, reasons for absence, and make-up learning sessions provided. CHWs will evaluate receipt of the intervention during each session by asking questions and generating discussion to assess the degree to which participants understand each learning session. CHWs will also evaluate the enactment of intervention skills during the 3 monthly continued support sessions by discussing diet changes, physical activity, number of steps taken per day, and stress management. RAs will also assess enactment during exit interviews with participants.

### Measures

Data collection will occur in a private location in the participant’s home or a private room at one of the churches (approximately 90 min), preferably in the morning after an overnight fast. All instruments are available in English and Spanish. Bilingual RAs blinded to study group assignment will collect data. The Principal Investigator will train RAs using a standardized system for collecting physiologic measures and questionnaire data. We will develop a training manual for administering questionnaires and biospecimens and other physiologic data. RAs will receive copies of the training manual. To ensure inter-rater reliability, RAs will be trained and tested for inter-rater reliability prior to each data collection on height, weight, waist circumference, blood pressure, and arterial stiffness. Questionnaires will be read to participants by a bilingual RA. Table 2 outlines the variable and measures being used in the study and measurement times. Data will be collected at Time 1 (0 months [baseline]), Time 2 (6 months [completion of the intervention]), and Time 3 (12 months [after 6 months with no contact from the study staff]). All instruments will be re-evaluated in this pilot study for future use in a larger trial.

**Table 2.** Summary of variables, measures, and time points

Variables and measurements	0	6	12	Alpha, ICC, *κ*
**Feasibility**				–
^a^Process evaluation checklist (bi-monthly)	–	–	–	
^a^Screening and enrollment logs	X	X	X	
^a^Intervention attendance	X	X	X	
^a^Data collection attendance	X	X	X	
Exit interview			X	
**Health history questionnaire**	X	X	X	–
**Sociocultural environmental**				
Demographic questionnaire (e.g., age, education, occupation)	X			–
Acculturation questionnaire (e.g., years in the US, language)	X			–
Everyday Discrimination Scale [79]	X			> .74
Brief Resilience Scale [80]	X			.83
Perceived Stress Scale-4 [81, 82]	X	X	X	.84–.88
**Built environment**				
Neighborhood Risk Assessment	X	X	X	–
Physical Activity Resource Assessment	X	X	X	–
**Behavioral factors**				
Food Behavior Checklist	X	X	X	–
Physical activity: Accelerometer for 7 days (24 h/day)	X	X	X	–
Global Physical Activity Questionnaire [83, 84]	X	X	X	*r* = 0.40^b^
Adapted Women’s Health Initiative Insomnia Rating Scale [85–87]	X	X	X	.71–.86
Eating Self-Efficacy Scale [88]	X	X	X	.88–.94
Exercise Self-Efficacy Scale [89–91]	X	X	X	.86–.92
**Biological outcomes (primary outcomes)**				
Systolic and Diastolic Blood Pressure [92]	X	X	X	*κ* = 0.74
Fasting glucose [93]	X		X	ICC = 0.98
Fasting lipid profile [93]	X		X	ICC = 0.95
Carotid-femoral pulse wave velocity [94]	X		X	ICC = 0.94
**Biological outcomes (secondary outcomes)**				
Height, weight, BMI (secondary outcomes)	X	X	X	–
Waist circumference [95]	X	X	X	ICC = 0.99
Hair cortisol and hsCRP	X		X	–
Vasomotor symptom questionnaire	X	X	X	–

Note: *0* baseline (T1); *6* 6 months (T2, completion of Phase I and Phase II intervention), *12* 12 months (T3, after 6 months of maintenance), *ICC* Intraclass correlation coefficient, *κ* kappa

^a^Completed by lead research assistant

^b^Correlation with accelerometer data

#### Primary Outcomes to be considered for main trial

##### Arterial stiffness

Pulse wave velocity (PWV), a measure of arterial stiffness will be measured in the central arterial region (carotid-femoral PWV) using an oscillometric device (Vicorder, Skidmore Industries, UK). This is a non-invasive approach that has been shown to be relatively independent of operator skills, with reproducible results [94, 96]. PWV is calculated as the time that the pulse wave takes to travel a definite distance along the vasculature (distance/transit time). As in prior studies [94, 97], we will measure the distance between the suprasternal notch and mid-upper thigh cuff to determine the distance traveled between pulse sites. Transit time is the time delay between the feet of the proximal and the distal pulse pressure waves. To assess transit time, pulse pressure waves will be captured using a 10-cm-wide cuff around the participant’s right upper thigh to detect the femoral pulse, and a 3-cm cuff around the neck to detect the right carotid pulse. A minimum of three cfPWV readings will be taken for each participant. The average of the closest two cfPWV readings (within 0.3 m/s) for each participant will be used in the analysis.

##### Blood pressure

RAs will measure systolic and diastolic blood pressure using a digital automatic blood pressure device (HEM-907/XL, Omron Healthcare) with the participant seated with both feet on the ground, legs uncrossed, following 3 to 5 min of sitting quietly and adopting a relaxed posture. Blood pressure measurements will be taken in the right arm, unless the blood pressure is known to be higher in that arm, or the presence of an anomaly or other circumstance prohibiting the use of the right arm. The average of 2 sequential readings will be used in the analyses.

##### Fasting lipids and glucose

Phlebotomy will be performed after an overnight fast, preferably in the morning. Participants will be seated quietly for 5 min immediately prior to venipuncture. Samples will be placed on ice and transferred to the Biobehavioral Laboratory at the School of Nursing at The University of North Carolina at Chapel Hill where they will be stored at 2–8 °C. Serum measures of total cholesterol, high-density lipoprotein cholesterol, triglycerides, and glucose will be analyzed using the Alere Cholestech LDX System. The Alere Cholestech LDX® Analyzer measures total cholesterol, HDL cholesterol, and glucose by an enzymatic method [93]. Low-density lipoprotein cholesterol will be calculated using the Friedewald equation [98].

#### Health behaviors

A bilingual RA will collect information on nutrition, physical activity, and sleep. All questionnaires have been validated in English and Spanish. We will use the University of California (UC) Cooperative Extension Food Behavior Checklist to evaluate eating habits [99–102], which has shown good validity and internal consistency (Cronbach’s alpha = 0.6–0.8) among Latinas. This sixteen-item questionnaire assesses overall diet quality, fruit and vegetable intake, milk intake, fat and cholesterol intake, and food security. Each question is accompanied by a visual representation to facilitate administration among individuals with limited literacy. A healthy diet pattern will be identified by calculating the number of healthy diet components participants endorse (total score 0–15). Food security will be assessed by asking, “Do you run out of food before the end of the month” with a 4-point response (no; yes, sometimes; yes, often; yes, always).

We will assess self-reported physical activity in a typical week using an interviewer-administered, modified Global Physical Activity Questionnaire developed by the World Health Organization (WHO; www.who.int/chp/steps/ GPAQ/en). The validity and reliability of this instrument have been previously reported [83, 84]. This sixteen-item questionnaire elicits information on physical activity in three settings (work, travel to and from places, and recreational activities). Participants will be asked to think about activities that last at least 10 min in a typical week. The questionnaire assesses the number of hours/day and days/week that participants engaged in physical activity. Self-reported moderate physical activity is calculated by summing the minutes in moderate activity across the three settings. Self-reported moderate to vigorous physical activity will be calculated by adding minutes per day in moderate and vigorous activity from work and leisure.

In addition to self-report, accelerometry will be measured for 7 days in perimenopausal Latinas at baseline (Time 1), 6 months (Time 2), and 12 months (Time 3) using an ActiGraph GT3X+ accelerometer worn on the wrist to track physical activity. Accelerometers can estimate the intensity, frequency, and duration of physical activity [103]. Approximately 7 days of monitoring, inclusive of Saturday and Sunday, with a minimum wear of 10 h is required to estimate physical activity in adults [104] reliably. Education of proper use and purpose of the watch will be provided to participants, and they will be requested to wear them for 24 h a day for 7 consecutive days. To promote greater compliance with the use of the accelerometer, all participants will be provided a phone number to call if they have questions. Participants will meet the RA at a community site to return the accelerometer after 1 week of use.

Information on sleep will be collected using a 4-item questionnaire adapted from the Women’s Health Initiative Insomnia Rating Scale [85, 86], which has been used in the Study of Women’s Health Across the Nation [105]. The scale consists of questions about difficulties experienced during sleep initiation, sleep maintenance, early morning awakening, and sleep quality during the past 2 weeks. Participants are asked to rate difficulties from 0 (no experience of the problem in the last 2 weeks) to 4 (experiencing the problem more than 5 times a week in the last 2 weeks). The overall quality of nighttime sleep will be rated from 0 (very restful) to 4 (very restless). Scores are summed for a maximum total of 16, with lower scores indicating poorer sleep.

#### Self-efficacy

This study will assess nutrition and exercise self-efficacy. The Eating Self-Efficacy Scale (Cronbach’s alpha 0.88–0.94) will be used to measure nutrition efficacy in perimenopausal Latinas ages 40 to 55 years [88]. Participants will respond to this 25-item tool to rate their difficulty in regulating eating from 1 (no difficulty) to 6 (very difficult) on two subscales, negative affect eating, and socially acceptable eating circumstances. Negative affect eating is the propensity to eat in response to negative emotions, while socially acceptable eating is associated with the overconsumption of food at social settings such as family events or holidays. Answers are summed reverse scored (total score 25–150), with a lower score reflecting greater difficulty controlling eating behaviors. We will use the Exercise Self-Efficacy Scale (Cronbach’s alpha 0.86–0.92) to assess participants’ beliefs in their ability to exercise [89]. The scale consists of 9 items that ask participants to rate their confidence to exercise (ranging from not confident at all = 0, to certainly confident can do = 10) when faced with barriers. Scores are summed and then divided by 9 to calculate a total score (0 to 10), with higher scores indicating greater exercise self-efficacy.

#### Anthropometric measures

Height and weight will be measured in a private room in light clothing without shoes. Height will be measured twice to the nearest 0.1 cm using a stadiometer, calibrated in 1/8-cm (cm) intervals. Weight will be measured to the nearest 0.1 kg using a Tanita WB-110A Digital Scale. Body mass index (BMI) will be computed as weight divided by the square of height (kg/m^2^) using a calculated field in the RedCap database. Based on BMI, we will determine if participants are normal weight (BMI < 25 kg/m^2^), overweight (BMI 25–29.9 kg/m^2^), or obese (BMI ≥ 30 kg/m^2^). RAs will measure waist circumference (at the umbilicus) using a Lufkin tape following the Multi-Ethnic Study of Atherosclerosis protocol [95, 106]. Measures will be taken twice and averaged.

#### Inflammatory and stress biomarkers

High-sensitivity C-reactive protein (hsCRP), a marker of inflammation [107], will be assessed by a blood draw. Samples will be placed on ice and transferred to the Biobehavioral Laboratory at the School of Nursing at The University of North Carolina at Chapel Hill where they will be stored at 2–8 °C. All samples will be analyzed using quantitative sandwich enzyme-linked immunosorbent assay (ELISA) (MyBiosource Inc, CA). Quality control assays assessing reproducibility identified the intra-assay CV (%) and inter-assay CV (%) as less than 15%.

#### Vasomotor symptoms

Vasomotor symptoms (hot flashes and night sweats) will be assessed via questionnaire as in the multi-ethnic Study of Women of Women’s Health Across the Nation [108]. Women will be asked two questions that separately assess how often they have experienced: (1) hot flashes and (2) night sweats in the past 2 weeks (not at all, 1–5 days, 6–8 days, 9–13 days, every day). Responses may be categorized as none, 1–5 days, and ≥ 6 days for analysis if cell sizes are small in the higher symptom categories.

#### Health history, sociocultural factors, built environment

Participants will complete a demographic data sheet and health history questionnaire. The sociodemographic data sheet will include information on age, Latino sub-group/nationality, marital status, employment status, highest education level, income, and health insurance. The health history questionnaire will collect information on tobacco use, asthma, cancer, thyroid disorders, diabetes, hypertension, hypercholesterolemia, current medications (e.g., anti-hypertensives, insulin, anticoagulants, antidepressants, hormonal therapies), and family history of CVD. To assess the history and frequency of tobacco use, participants will respond to three items, (1) “Have you ever smoked at least 100 cigarettes in your entire life;” (2) “Do you smoke cigarettes now?”; and, if they are current smoker, (3) “How many cigarettes on average do you smoke per day now?” In terms of reproductive history, women will be asked about their age at menarche, parity, complications during pregnancy (high blood pressure, diabetes, preeclampsia), date of last menstrual period, hysterectomy status, and menstrual patterns (regularity, duration, frequency, flow, pain during periods).

We will gather information on the sociocultural environment using interviewer-administered questionnaires, which will include the Perceived Stress Scale-4 (PSS-4) [81, 82], the Everyday Discrimination Scale [79], and the Brief Resilience Scale [80]. The PSS-4 is a 4-item questionnaire that uses a 5-point Likert scale to measure the degree to which participants appraise certain situations as stressful in the past month (never = 0, almost never = 1, sometimes = 2, fairly often = 3, and very often = 4); a raw score is calculated by summing the responses, providing a range of 0 (low stress) to 16 (high stress). This scale has good internal consistency (Cronbach’s alpha = 0.82) [109] and has been previously used to measure stress in perimenopausal women [77].

We will use the Everyday Discrimination Scale to assess how often participants encounter discriminatory treatment in their day-to-day lives (never = 0, less than once a year = 1, a few times a year = 2, a few times a month = 3, at least once a week = 4, and almost every day = 5). The Everyday Discrimination Scale has demonstrated good internal consistency (Cronbach’s alpha > 0.74) [79], test-retest, and convergent and divergent validity. Scores are summed, resulting in a possible overall score of 0 to 45; higher scores suggest greater reported experiences of discrimination.

The Brief Resilience Scale assesses a person’s ability to bounce back or recover from stress. It contains statements such as “I tend to bounce back quickly after hard times” and is scored using a 5-point Likert scale (strongly disagree = 1, disagree = 2, neutral = 3, agree = 4, strongly agree = 5). Scores are summed and averaged, providing a range of 1 to 5, with higher scores indicating greater resilience. The Brief Resilience Scale has shown adequate reliability (Cronbach’s alpha = 0.83; intraclass coefficient = 0.69) and validity [80].

Information on the built environment, including the availability of grocery stores and supermarkets, physical activity resources, and public transportation, will be gathered using a community and physical activity research assessment instrument. Participants will be asked to rate (poor, fair, good, or excellent) the availability of recreational areas (e.g., local parks, gyms, and safe spaces to walk) in the county/neighborhood where they live. Information on the frequency and duration of visits to recreation areas will also be collected as well as details of the condition of the location and availability of any programs. A community risk assessment tool will also be used to gather information on the participant’s perception of the neighborhood (i.e., visual appearance, safety, social cohesion). Prior studies have shown that neighborhood built and social environments are associated with health-related behaviors [33, 36].

### Process evaluation guide

The Lead RA will observe two randomly selected interventions sessions per month using a guide to assess whether sessions are being conducted according to the intervention protocol and if the sessions are engaging the participants. The Lead RA will also use the process evaluation guide to determine if there are any issues or concerns that need to be addressed based on observations and feedback from participants.

### Exit interview

A semi-structured interview, lasting approximately 15 min, will be conducted at Time 3 data collection with each participant. Exit interviews will consist of 12 open-ended questions to elicit participants’ opinions of the intervention and the time and setting of sessions (intervention group), study procedures and measures, and suggestions for improvement of the intervention and study protocol. Sample questions included, “what did you enjoy and find helpful about participating in the program” and “what is your advice to us about how to make it [the program] better?”

### Data management

Participants will be assigned a study subject number as an identifier (ID). Data will be recorded on forms on which the only identifier is the subject ID code. All data will be entered into REDCap by RAs with built-in range checks and skip patterns. A different RA will check data entry against the raw data for inconsistencies and correct the entry as needed. Data will be verified and stored in a secure server. Data will undergo range, consistency, and outlier checks. All data decisions will be discussed during research team meetings and recorded in a codebook with an audit trail. REDCap data will be exported into SAS Version 9.4 for data analysis.

### Data analysis

#### Aim 1

Using descriptive statistics, we will summarize the following: 1) the number of women screened; 2) enrollment rate (proportion of eligible women who enrolled after screening); 3) retention rate for enrolled participants (proportion of women who completed the study); and 4) attendance for women in the intervention group (percent of sessions attended). We will measure fidelity of the intervention (≥ 80% attendance, delivering > 80% of content) using the process evaluation guide and assess participant satisfaction with the study and the intervention using the exit interview.

#### Aim 2

An intent-to-treat analysis will be used in which all participants are analyzed according to their initial randomized assignment. Because the random assignment was by the group and not individually, we will examine closely potential systematic differences between the two groups, comparing baseline characteristics using Student’s *t* test or Mann-Whitney test for continuous variables and chi-square or Fisher’s exact test for categorical variables. The use of repeated measures alleviates this potential bias by assessing within-person change rather than post-only measures, which offers some degree of control for baseline differences. To assess the impact of the intervention, we will separately assess the reduction of biologic CVD risk factors and increase in health behaviors and self-efficacy using generalized estimating equations for categorical outcomes (logistic mixed-effects models focusing on group proportions) and linear mixed-effects models for continuous outcomes. Models will include data collection time point (Time 1, 2, or 3), group assignment, and the interaction between the two (time × group). We will adjust models for baseline covariates associated with the outcome measures at *p* < 0.05 [110, 111]. We will perform sensitivity analyses to estimate the effect when excluding women on anti-hypertensive, lipid-lowering, or diabetes medications. We will re-fit the models using multiple imputations to assess the impact of ignoring missing data.

### Data monitoring, safety, and adverse events

Oversight and monitoring of the conduct and progress of the study will be provided by the steering committee and the Data Safety and Monitoring Board (DSMB). The principal investigator, steering committee members, and/or study staff will conduct medical monitoring for unanticipated problems, adverse events, and serious adverse events and record and report them to the Institutional Review Board. Study progress and safety will be reviewed monthly. The principal investigator will notify the DSMB, Institutional Review Board, and sponsor regarding conduct medical monitoring for unanticipated as appropriate.

Detailed information concerning adverse events and serious adverse events will be collected and evaluated throughout the trial. Participants will be queried about the occurrence of adverse events at each visit. They will also be instructed to phone at any time during the study with an AE. The principal investigator, steering committee, and/or study staff will report all adverse events and serious adverse events to the DSMB. The DSMB will review all adverse events, serious adverse events, and other interim safety data and will provide a report to the PI and the IRB. An incident report entered into the data monitoring system within 48 h of learning of the serious adverse event.

## Discussion

CVD, including coronary heart disease and stroke, is the leading cause of death among Latinas [112]. Despite being at greater risk for CVD, only 34% of Latinas are aware that CVD is the leading cause of death in women [5]. Although several national organizations have developed initiatives to raise understanding about CVD among Latinas, as yet Latinas remain considerably less likely to meet physical activity and dietary guidelines than non-Hispanic white women, particularly during perimenopause [7]. Perimenopause is marked by dramatic changes in sex hormone levels, which may adversely affect CVD risk factors (i.e., body fat, insulin secretion, lipoprotein levels) [8, 113]. Thus, perimenopause is a critical window for CVD prevention among Latinas.

This will be the first behavioral intervention to focus on reducing biologic CVD risk among perimenopausal Latinas. Although interventions among perimenopausal women have focused on CVD risk education and physical activity, this study will use a multi-component intervention delivered by CHWs that includes stress management and coping skills training in addition to interactive education sessions and group physical activity to reduce biologic CVD risk. Furthermore, this pilot study will include longitudinal measures of arterial stiffness assessed by cfPWV, a non-invasive measure predictive of CVD [16] that has not been incorporated in prior interventions among Latinas.

This study will provide preliminary data on the feasibility and initial efficacy of a multi-component intervention (cardiovascular health education, physical activity, stress management, and coping skills training) among perimenopausal Latinas to (a) decrease biological CVD risk factors (arterial stiffness, blood pressure, lipids, blood glucose), (b) improve health behaviors and self-efficacy, and (c) improve adiposity, inflammatory and stress biomarkers, and vasomotor symptoms. The results of this pilot study will provide an assessment of the feasibility of the protocol and preliminary effect sizes to inform the development of a larger-scale trial. This study has the potential to greatly accelerate the acquisition of knowledge related to CVD risk among perimenopausal Latinas and the impact of behavioral interventions to reduce CVD risk in this underserved population. Furthermore, this study may provide insight into the associations between sociocultural, environmental, behavioral, and biological factors contributing to women’s overall cardiovascular health.

## Data Availability

Data sharing is not applicable to this manuscript since no datasets have been generated or analyzed to date. Data will be available after completion of the study upon reasonable request to the Principal Investigator, Dr. Yamnia I. Cortés.

## Abbreviations

BMI: Body mass index
cfPWV: Carotid-femoral pulse wave velocity
CHW: Community health worker
CVD: Cardiovascular disease
hsCRP: High-sensitivity C-reactive protein
ID: Identifier
PSS: Perceived stress scale
PWV: Pulse wave velocity
RA: Research assistant
SCSV: Su Corazón Su Vida
US: United States
